# The complete chloroplast genome of *Illicium verum* and comparative analysis with related species from Magnoliaceae and Illiciaceae

**DOI:** 10.3389/fgene.2024.1452680

**Published:** 2024-12-11

**Authors:** Yingying Cao, Yongxing Lai, Zhuxin Li, Shanshan Zhai, Yinghan Dai, Junyu Tao, Qing Wang, Ziheng Xu, Minjie Jiang, Li Yu, Jing Leng, Haibo Tang

**Affiliations:** ^1^ Guangxi University of Chinese Medicine, Nanning, China; ^2^ Guangxi Vocational University of Agriculture, Nanning, China; ^3^ Guangxi Key Laboratory of Translational Medicine for Treating High-Incidence Infectious Diseases with Integrative Medicine, Nanning, China; ^4^ Guangxi Zhuang Autonomous Region Engineering Research Center of Graphene Biomedical Application Technology, Nanning, China; ^5^ Key Laboratory of Characteristic Experimental Animal Models of Guangxi, Nanning, China; ^6^ Guangxi Health Commission Guangxi Key Laboratory of Molecular Biology of Preventive Medicine of Traditional Chinese Medicine, Nanning, China

**Keywords:** chloroplast genome sequence, comparative analysis, *Illicium verum*, phylogenetic relationship, Illiciaceae

## Abstract

*Illicium verum* (Illiciaceae), an ecologically significant endemic plant, predominantly grows in Guangxi, China, which is the primary region for its cultivation. This area accounts for more than 80% of the total cultivation and yield in China. Despite its importance, comprehensive studies on the chloroplast (cp) genome of *I. verum* are limited. In our research, we sequenced and analyzed the complete cp genome of *I. verum* and conducted a comparative analysis with nine related species from the families Magnoliaceae, Schisandraceae, and Illiciaceae. The cp genome of *I. verum* spans 143,187 base pairs (bp), comprising a large single copy (LSC) region of 100,868 bp, a small single copy (SSC) region of 20,235 bp, and two inverted repeats (IR) regions, each 11,042 bp in length. We identified 81 simple sequence repeats (SSRs) within this genome. The chloroplast genome contains 78 protein-coding genes, 8 ribosomal RNA (rRNA) genes, and 35 transfer RNA (tRNA) genes. Structurally, the IR regions exhibit greater similarity across different genera of Magnoliaceae and Illiciaceae compared to the LSC and SSC regions. Phylogenetic analysis revealed high homology between the cp genome of *I. verum* and those of *Illicium burmanicum*, *Illicium simonsii*, and *Illicium anisatum*. These findings suggest that the cp genome of *I. verum* may serve as a valuable genomic resource for elucidating the phylogenetic positions and relationships within the Illiciaceae family. This information will be instrumental for future taxonomic research on *Illicium* species and for advancing genomic studies of these plants.

## 1 Introduction

*Illicium verum Hook.f.,* (*Illicium verum*, Illiciaceae) is an aromatic evergreen tree that produces purple-red flowers and star-shaped fruit with an anise scent. It is classified within the division Magnoliophyta, class Magnoliopsida, subclass Magnoliidae, order Austrobaileyales, and family Illiciaceae (China EBoFo, 2004). Initially, *Illicium* was placed in the Magnoliaceae family in early taxonomic literature, but later it was reclassified into the Illiciaceae family by Smith based on floral morphology and vegetative anatomy (AC, 1947). Its fruit, also known as star anise, is not only a significant component of traditional Chinese medicine but also a commonly used spice.

*Illicium verum*, recognized in traditional Chinese medicine as Anisi Stellati Fructus or Bajiaohuixiang, is renowned for its medicinal properties, particularly in China. It has been utilized in various formulations, including crude drugs, powders, and essential oils. The use of *I. verum* is documented as far back as the Ming Dynasty in the “Compendium of Materia Medica” (Li, 1596). The Chinese Pharmacopoeia describes Anisi Stellati Fructus as having properties that warm yang, dispel cold, and regulate the flow of Qi, thereby alleviating pain or symptoms of the common cold. Clinically, it is applied to treat conditions such as abdominal colic, vomiting, and lower back pain. Furthermore, the crude fruits or their powdered forms have been integrated into traditional teas to mitigate nervousness, insomnia, and to serve as a sedative (Wang et al., 2011).

*Illicium verum* has been cultivated in the Zuoyou River valley in Guangxi Province since the Ming Dynasty in ancient China. With the development of China’s Maritime Silk Road and the expansion of overseas trade, Fujian Province and Guangdong Province have also become production and sales locations for *I. verum*. These provinces, located south of 25° north latitude, have a subtropical climate in hilly and mountainous regions with warm winters and cool summers, resulting in good fruit quality. Guangxi Province has maintained its status as a geo-authentic crude drug-producing area of *I. verum*, particularly in Longzhou, Ningming, Napo, and other counties in the west and south of Guangxi (Qi, 1995). In December 2020, the Administration of Traditional Chinese Medicine of Guangxi Zhuang Autonomous Region and other departments included *I. verum* in the Guangxi authentic medicinal materials “Ten Herbs of Guangxi,” initiating key development and utilization efforts (Li, 2023).

Because there are too many similarities in the phenotypes (tree shape, branch, stem, leaf, flower, pollen, fruit) of the Illiciaceae plants, the differences are not always obvious (Zhang et al., 2017b). This can make it difficult to identify the species of Illiciaceae, even for professionals. Unfortunately, there is no perfect method for identification. The compounds contained in the plants vary greatly, leading to significant differences in efficacy and toxicity. As a result, there have been cases of ingestion poisoning. Effectively identifying medicinal materials and counterfeit products is a challenging problem. The U.S. Food and Drug Administration (FDA) has developed a GenomeTrakrCP database, a public database of chloroplast genome sequences for plant flavors, plant dietary supplements, etc., including Illiciaceae, to help identify plant species in foods and dietary supplements. Includes plants used as food and dietary supplements, toxin producers, common contaminants and adulterants, and their relatives (Chen et al., 2023; Cui and Fu, 2001; Li and Liu, 2011; Vermaak et al., 2013; Zhang et al., 2017b).

Typically, taxonomic research relies on morphological evaluation, a method prone to environmental influences. Discerning various morphological traits in plants necessitates observations across their entire life cycle, imposing constraints on identifying species lacking comprehensive morphological data. In contrast, molecular identification, which utilizes DNA markers, offers enhanced precision and dependability due to the inherent genetic stability and resilience to external variables. Consequently, this approach is better suited for distinguishing closely related species. The chloroplast genome, generally spanning 100–150 kilobases and rich in evolutionary information, serves as an exemplary model for investigations into molecular biomarkers, phylogenetic analyses, evolutionary studies, and comparative genomics (Wu et al., 2010; Wang et al., 2016; Hong et al., 2017; Zhang et al., 2017a; Zhang et al., 2017b).

Little molecular research has been done on the Illiciaceae. This has hindered further studies of the molecular identification, phylogeny, and evolution of the genus. In this study, we report the sequence, assembly, annotation, and structural analysis of the *I. verum* chloroplast genome. The publication of more cp genomes from the Illiciaceae will help identify genetic variation through sequence comparison and provide new insights into evolutionary history and interspecific relationships. The study provides a basis for identifying medicinal properties, revising quality standards, and further development and utilization.

## 2 Materials and methods

### 2.1 Sampling, DNA extraction, sequencing, and assembly

*Illicium verum* samples were collected from the nursery at Guangxi University of Chinese Medicine, with the specimen accession number Bankit2753337. These samples were promptly stored at −80°C. Genomic DNA was extracted from the leaves using a modified cetyl trimethyl ammonium bromide (CTAB) protocol (Doyle and Doyle, 1987). Once the DNA quality was confirmed, shotgun sequencing libraries with an insert size of 250 base pairs were prepared according to the standard protocol provided by Illumina Inc. (San Diego, CA, United States). Sequencing was conducted on an Illumina HiSeq platform by Genesky Biotechnologies Inc. (Shanghai, China), employing the PE150 strategy (Feng et al., 2022; Shen et al., 2022).

Quality control of the raw sequencing data was performed using FastQC software, version 0.11.8 (Dierckxsens et al., 2017). High-quality clean reads were generated by eliminating adapters and low-quality sequences from the raw data with Trimmomatic, version 0.35 (Bolger et al., 2014). The chloroplast genome of *I. verum* was assembled using the metaSPAdes algorithm (Bankevich et al., 2012), with the reference being the previously sequenced *I. verum* chloroplast genome (NCBI accession number: NC_034689). To confirm the accuracy of the genome, the Illumina reads used for assembly were mapped back to the cp genome using Genious 8.1 (Kearse et al., 2012).

### 2.2 Annotation of the *I. verum* cp genome

The chloroplast genome of *I. verum* was annotated using the CpGAVAS pipeline (Liu et al., 2012). The annotated genome sequence has been deposited in GenBank with the accession number OR668891. Validation of annotated genomes using DOGMA (Dual Organellar Genome Annotator) software (Jansen et al., 2005). Visualization of the circular gene map was achieved using OGDRAW version 1.2 (Greiner et al., 2019), accessed on 12 September 2021, via the website http://ogdraw.mpimp-golm.mpg.de/. The relative synonymous codon usage (RSCU) analysis was conducted using CodonW, version 1.4.4 (http://codonw.sourceforge.net/, accessed on 15 September 2021).

### 2.3 Chloroplast genome comparison

To verify the possibility of genomic differentiation, we downloaded the chloroplast genomes of eight closely related species from Illiciaceae, Schisandraceae and Magnoliaceae from GenBank, including *I. anisatum L.*, *I. henryi Diels*, *I. lanceolatum A.C.Smith*, *Kadsura heteroclita (Roxb.) Craib*, *Liriodendron chinense (Hemsl.) Sarg*, *Magnolia maudiae (Dunn) Figlar Magnolia ovata (A.St.-Hil.) Spreng*, and *Schisandra henryi Clarke.*, and compared them using mVISTA in the shuffle-LAGAN mode (Frazer et al., 2004). The expansion and contraction of the IRs were conducted using IRSCOPE (Amiryousefi et al., 2018). To identify the hypervariable regions within the cp genome of Illiciaceae species, the cp genomes of *I. verum* (OR668891, OK377288), *I. henryi* (NC-034699), *I. lanceolatum* (OL802931) and *I. anisatum* (KY085919) were aligned using MEGA7. The alignment was manually adjusted using Se-Al 2.04, and the nucleotide diversity (Pi) along the cp genome was calculated using DnaSP version 5 software (Librado and Rozas, 2009) with sliding window analysis.

### 2.4 Identification of repeats

Tandem Repeats Finder (Benson, 1999) was used to identify repeat sequences. Then, the online microsatellite identification tool (MISA, available online: https://webblast.ipk-gatersleben.de/misa/) (Beier et al., 2017) was applied to predict cpSSRs with default parameters.

### 2.5 Phylogenetic analysis

To determine the phylogenetic relationships among Illiciaceae species, Maximum-likelihood (ML) phylogeny was built using MEGA 7 with 1000 bootstrap replicates (Kumar et al., 2016). An alignment of 53 cp genomic sequences was created using the MAFFT online version with default parameters.

## 3 Results

### 3.1 Genome features of *I. verum*

After filtering the raw sequencing data, we obtained a total of 646,898 high-quality paired-end reads. These reads comprised 96,506,157 bases, with 88.5% exceeding a quality score of Q30. The complete chloroplast (cp) genome of *I. verum* measures 143,187 base pairs (bp) in length and exhibits a guanine-cytosine (GC) content of 39.1%. The assembled genome achieved an average read coverage exceeding 700-fold.

**FIGURE 1:**
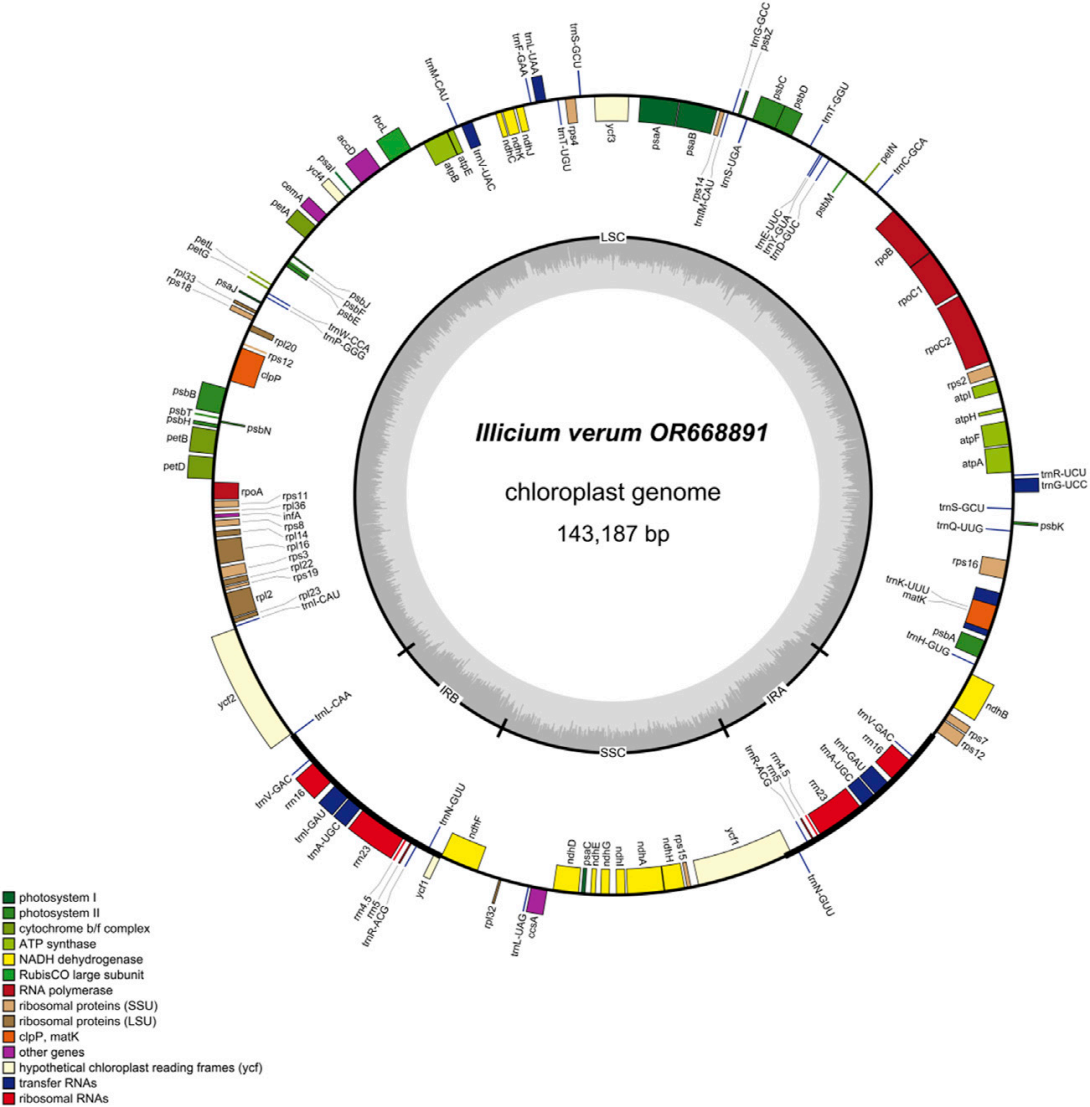
Chloroplast genome map of *I. verum*. Genes inside the circle are transcribed clockwise, and those outside are transcribed counterclockwise. Genes of different functions are color-coded. The darker gray in the inner circle shows the GC content, while the lighter gray shows the AT content.

The genome of *I. verum* displays a typical quadripartite structure, containing one large single copy (LSC; 100,868 bp) region, one small single copy (SSC; 20,235 bp) region, and two inverted repeat regions (IRs; 11,042 bp each) (Figure 1). The DNA G + C contents of the LSC, SSC, and IR regions and the whole genome are 38%, 33.9%, 49.1%, and 39.1%, respectively, which is also similar to the chloroplast genomes of other *I. verum* (Table 1).

**TABLE 1:** Base composition in the cp genome of *I. verum*.

Region	Length	T(U)%	C%	A%	G%
Genome	143187	31	19.9	29.9	19.2
LSC	100868	31.7	19.4	30.4	18.6
SSC	20235	33.4	17.8	32.6	16.1
IRa	11042	26.3	26.6	24.7	22.5
IRb	11042	24.6	22.5	26.3	26.6
CDS^a^	68415	30.8	18.3	30	20.9
CDS, 1 position^b^	22805	23.2	19	30	27.8
CDS, 2 position^c^	22805	32	20.8	28.7	18.5
CDS, 3 position^d^	22805	37.2	15.2	31.3	16.3

^a^ CDS = Protein-coding regions.

^b^ 1 position = 1st base of codons.

^c^ 2 position = 2nd base of codons.

^d^ 3 position = 3rd base of codons.

In the *I. verum* cp genome, 121 functional genes were predicted, including 8 rRNA genes, 35 tRNA genes, and 78 protein-coding genes (Table 2). Cp genomes have 10 duplicated genes in the IR regions, including approximately 5 tRNA genes (tRNAs), 4 rRNA genes (rRNAs), and 1 protein-coding gene (PCGs) (Figure 1). The LSC region contains 68 protein-coding and 28 tRNA genes, while the SSC region contains 1 tRNA gene and 12 protein-coding genes.

**TABLE 2:** Gene contents in the *I. verum* cp genome.

Category for genes	Group of genes	Name of genes
Transcription and translation-related genes	transfer RNAs	trnH-GUG, trnK-UUU, trnQ-UUG, trnS-GCU, trnG-UCC, trnR-UCU, trnC-GCA, trnD-GUC, trnY-GUA, trnE-UUC, trnT-GGU, trnS-UGA, trnG-GCC, trnfM-CAU, trnS-GCU, trnT-UGU, trnL-UAA, trnF-GAA, trnV-UAC, trnM-CAU, trnW-CCA, trnP-GGG, trnI-CAU, trnL-CAA, trnV-GAC, trnI-GAU, trnA-UGC, trnR-ACG, trnN-GUU, trnL-UAG, trnN-GUU, trnR-ACG, trnA-UGC, trnI-GAU, trnV-GAC
	RNA polymerase	rpoC1, rpoC2, rpoA, rpoB
	ribosomal proteins(SSU)	rps2, rps3, rps4, rps7, rps8, rps11, rps12, rps14, rps15, rps16 rps18, rps19,
	ribosomal proteins(LSU)	rpl2, rpl14, rpl16, rpl20, rpl22, rpl23, rpl32, rpl33, rpl36
	Translational initiation factor	infA
	ribosomal RNAs	rrn4.5, rrn5, rrn16, rrn23
Photosynthesis-related genes	NADH dehydrogenase	ndhA, ndhB, ndhC, ndhD, ndhE, ndhF, ndhG, ndhH, ndhI, ndhJ, ndhK
	photosystem I	psaA, psaB, psaC, psaI, psaJ
	photosystem II	psbA, psbB, psbC, psbD, psbE, psbF, psbH, psbJ, psbK, psbM, psbN, psbT, psbZ
	cytochrome b/f complex	petA, petB, petD, petG, petL, petN
	RubisCO	rbcL
	ATP synthase	atpA, atpB, atpE, atpF, atpH, atpI
	hypothetical chloroplast	ycf1, ycf2, ycf3, ycf4
Other genes	Maturase	matK
	Protease	clpP
	Envelope membrane protein	cemA
	Subunit of Acetyl-CoA carboxylase	accD
	C-type cytochrome synthesis gene	ccsA

Within the genome, 20 genes are identified to harbor introns, comprising 12 protein-coding genes and 8 transfer RNA (tRNA) genes as detailed in Table 3. Specifically, 18 genes-encompassing 10 protein-coding genes and 8 tRNA genes-feature at least one intron. Notably, the genes *ycf3* and *clpP* are distinguished by the presence of two introns. Among the intron-containing genes, the trnK-UUU gene is notable for having the largest intron, measuring 2541 base pairs (bp), contrasting with the trnL-UAA gene, which contains the smallest intron at 517 bp. Additionally, the *rps12* gene is recognized as a trans-splicing gene, with its 5′ end located in the large single copy (LSC) region and the 3′ end situated in the inverted repeat (IR) region.

**TABLE 3:** Genes with introns in the *I. verum* cp genome.

Genacte	Location	Exon Ⅰ(bp)	Intron Ⅰ(bp)	Exon Ⅱ(bp)	Intron Ⅱ(bp)	Exon Ⅲ(bp)
rps12	LSC	232	526	26	0	0
ndhB	LSC	721	707	758	0	0
trnK-UUU	LSC	37	2541	37	0	0
rps16	LSC	40	833	221		
trnG-UCC	LSC	24	720	48		
atpF	LSC	145	811	416		
rpoC1	LSC	453	734	1617		
ycf3	LSC	124	742	230	756	153
trnL-UAA	LSC	35	517	50		
trnV-UAC	LSC	39	602	37		
clpP	LSC	71	798	294	639	244
petB	LSC	6	733	642		
petD	LSC	8	725	526		
rpl16	LSC	9	1015	402		
rpl2	LSC	397	665	431		
trnI-GAU	RepeatB	42	935	35		
trnA-UGC	RepeatB	38	800	35		
ndhA	SSC	553	1053	542		
trnA-UGC	RepeatA	38	800	35		
trnI-GAU	RepeatA	42	935	35		

### 3.2 Bias of codon usage

**FIGURE 2:**
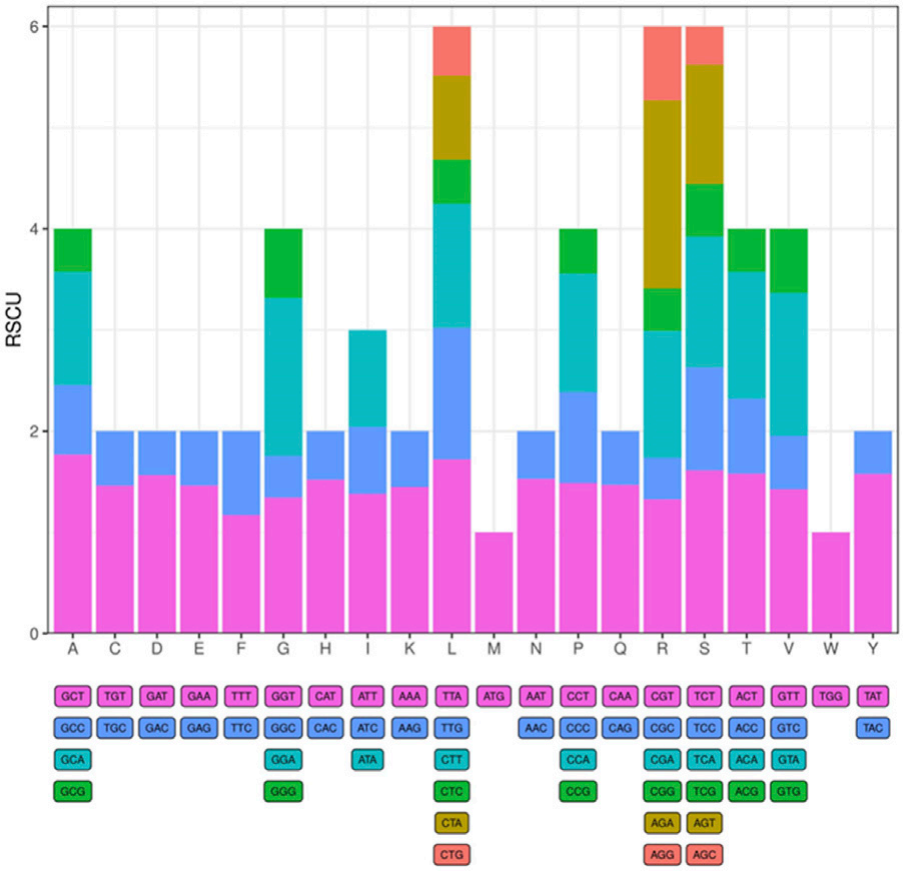
Codon content of 20 amino acids and stop codons in all protein-coding genes of the *I. verum* chloroplast genome. The codons are represented by different colors in the histogram.

The tRNA and protein-encoding gene sequences of the *I. verum* cp were analyzed, and the codon usage frequency of the cp genome of *I. verum* was inferred and summarized. A total of 22,727 codons represent the coding ability of *I. verum* (Figure 2; Supplementary Table S1), of which 2,319 codons code for leucine (10.2%), and 268 codons code for tryptophan (1.2%), which are the most and least common amino acids in the cp genome of *I. verum*, respectively. Most of the codons ending with A or T were presented as RSCU >1 (Supplementary Table S1).

### 3.3 Comparative analysis of genomic structure

**FIGURE 3:**
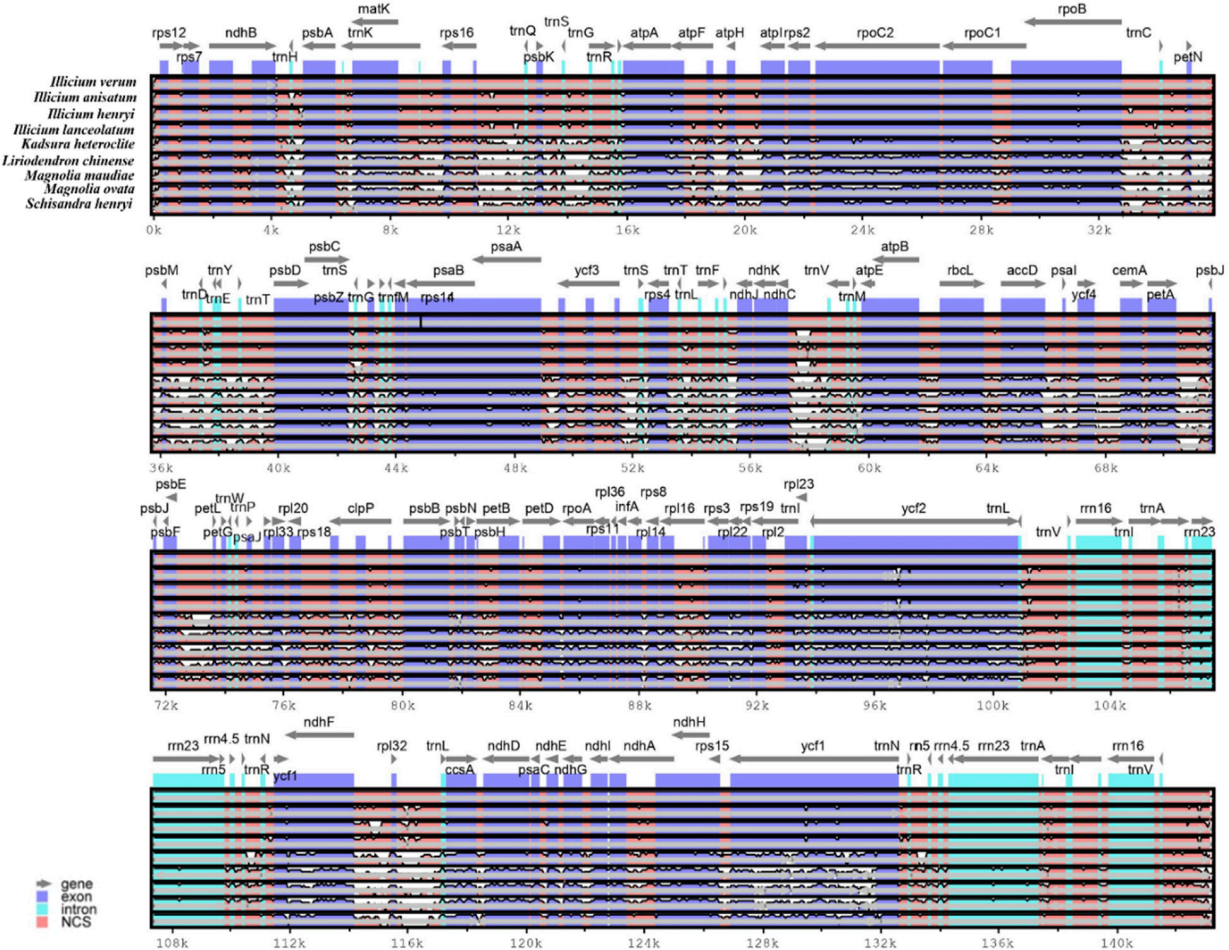
Comparison of the cp genome sequences of nine plants. Comparison of the cp genome sequences of *I. verum* (NC-034689), *Illicium anisatum* (KY085919), *Illicium henryi* (NC-034699), *Illicium lanceolatum* (OL802931), *Kadsura heteroclite* (NC-057266), *Liriodendron chinense* (MK887904), *Magnolia maudiae* (NC-047409), *Magnolia ovata* (MT682825) and *Schisandra henryi* (MH394370) generated with mVISTA. Gray arrows indicate the position and direction of each gene. Red and blue areas indicate the intergenic and genic regions, respectively. The vertical scale indicates the percentage of identity, ranging from 50% to 100%.

The complete cp genome sequence of *I. verum* was compared with *I. anisatum*, *I. henryi*, *I. lanceolatum*, *K. heteroclita*, *L*. *chinense*, *M*. *maudiae*, *M. ovata* and *S. henryi* has the largest cp genome (Figure 3). The results of this comparison revealed that the LSC and SSC regions are more divergent than the IR regions and that higher divergence is found in noncoding than in coding regions. The chloroplast genome sequence of *I. verum* was not significantly different from that of *I*. *anisatum*, *I*. *henryi*, and *I. lanceolatum*, but it was quite different from the five other plants.

**FIGURE 4:**
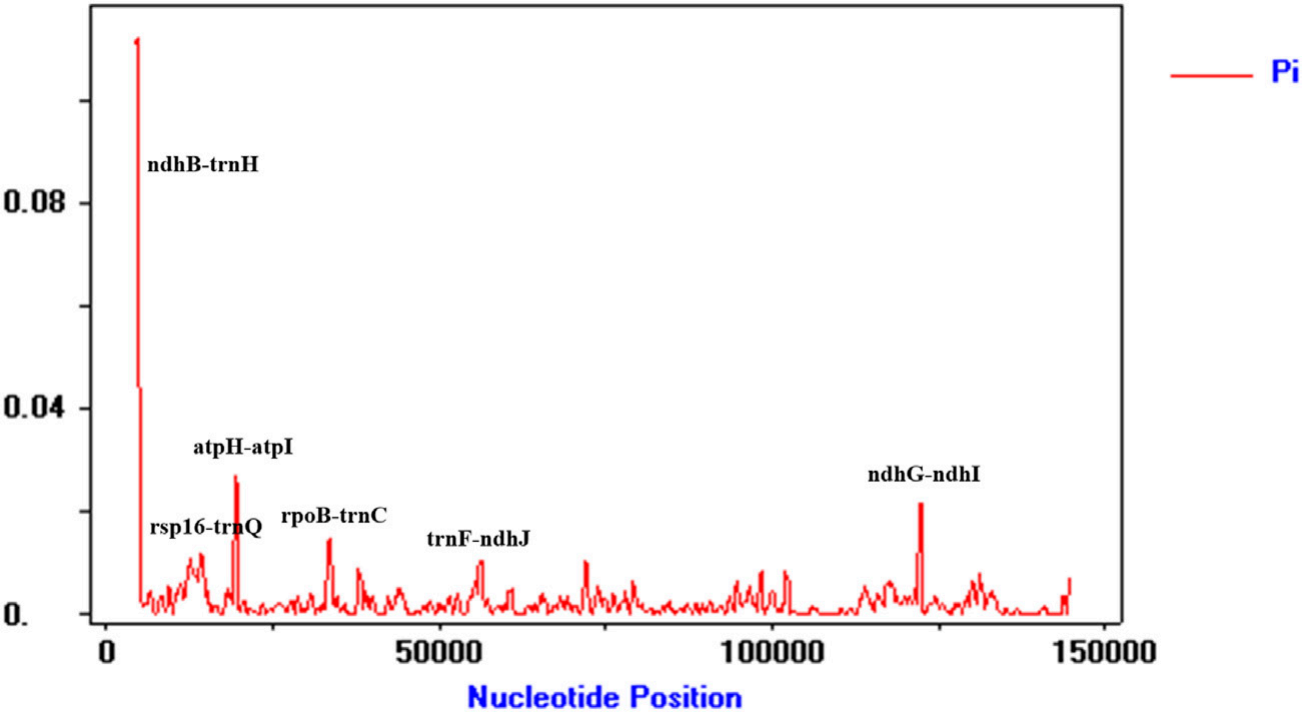
Chloroplast genome comparative analysis between *I. verum* (OR668891 and OK377288), *Illicium henryi* (NC-034699), *Illicium lanceolatum* (OL802931) and *Illicium anisatum* (KY085919). Sliding window plots of nucleotide diversity (Pi) across the complete cp genomes of Illiciaceae species (window length: 600 bp, step size: 200 bp). Y-axes: nucleotide diversity (Pi) of each window; X-axes: the position of the midpoint of a window.

To detect highly variable regions, we analyzed variable sites in the Illiciaceae chloroplast genome by sliding window analysis using the software DnaSP (Figure 4). The divergent loci (*ndhB-trnH*, *atpF-atpH, ndhG-ndhI*) had a Pi value greater than or equal to 0.02. The *ndhB-trnH, atpF-atpH* divergent loci were intergenic regions and were present in the LSC region, and *ndhG-ndhI* occurred in the SSC region, with none detected in the IR region. These results also confirmed that the LSC and SSC regions were less conserved than the IR regions.

### 3.4 Contraction and expansion of the IR regions

**FIGURE 5:**
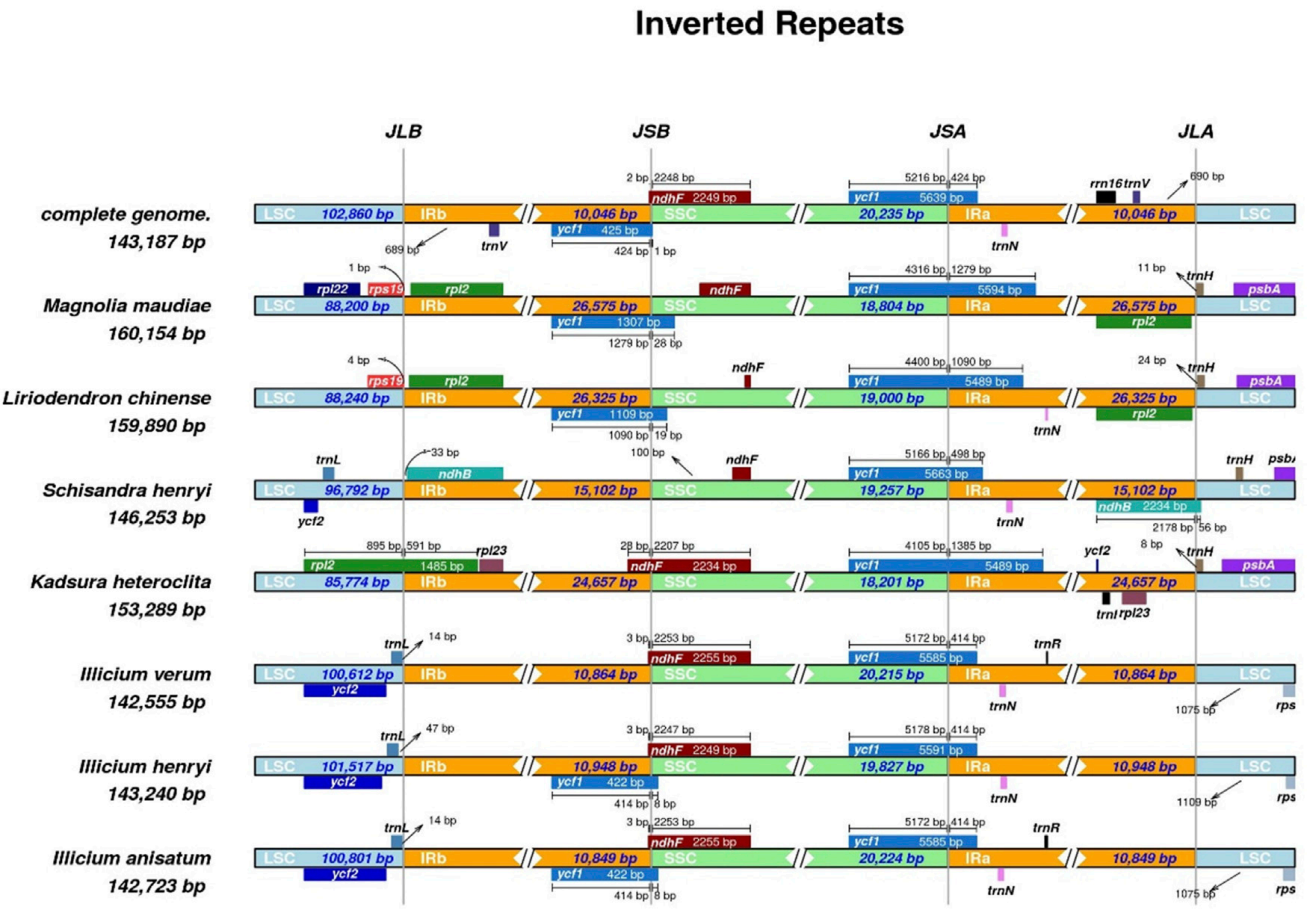
The junctions of IR/SC comparison among 8 plants. Arrows indicated the distance of the gene to the junctions, and the I-shaped line represented the length of the gene on either side of the junctions.

To further resolve the structural evolutionary history of the cp genomes of *I. verum*, we compared the IR/SSC and IR/LSC junctions across eight selected Magnoliaceae, Schisandraceae and Illiciaceae species, including *M*. *maudiae*, *L. chinense*, *S. henryi*, *K. heteroclita*, *I*. *henryi*, and *I*. *anisatum*. The results of the IRscope analysis are presented in Figure 5.

We observed a wide variability in the junction sites of these cp genomes. For example, the *ycf1* gene was located at the SSC/IRa junction in all the chloroplast genomes of different species. The length of the *ycf1* gene extending into the SSC region varied depending on the genome (*I. verum* OR668891, 5216 bp; *I. verum* OK377288, 5172 bp; *I*. *henryi* NC-034699, 5178 bp; *I*. *anisatum* KY085919, 5172 bp; *M*. *maudiae* NC_047409, 4316 bp; *L. chinense* MK887904, 4400 bp; *S. henryi* MH394370, 5166 bp; *K. heteroclite* NC-057266, 4105 bp); the IRa region includes 424, 414, 414, 414, 1279, 1090, 498, and 1385 bp of the *ycf1* gene. In the boundary region of JSB (IRb/SSC), except for *M*. *maudiae*, *L*. *chinense* and *S*. *henryi*, the boundaries of JSB (IRb/SSC) in the other five plants were located within the *ndhF* gene in the SSC region and expanded by 2–28 bp to the IRb boundary.

### 3.5 Long-repeat and SSR analysis

**FIGURE 6:**
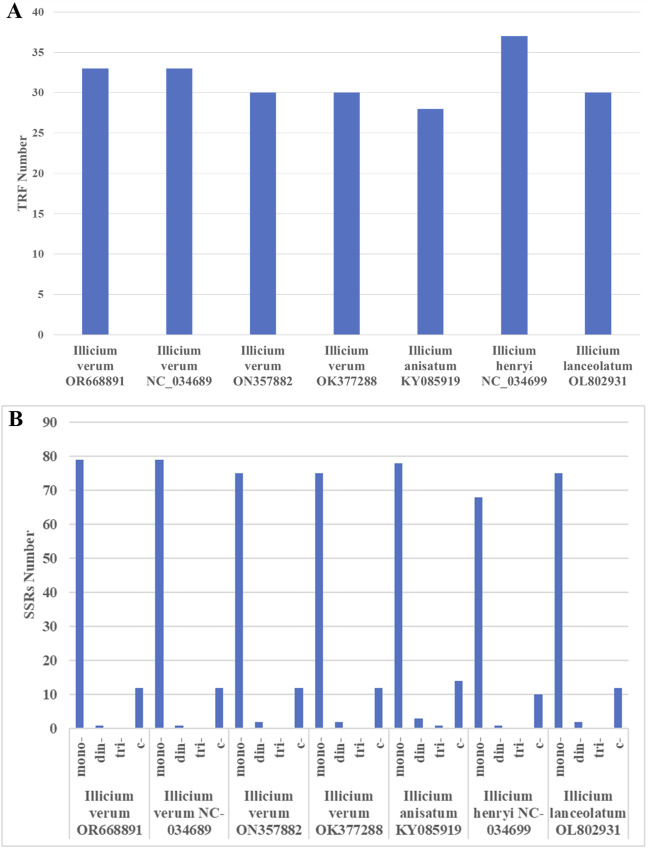
The type of long-repeat and SSRs in the seven Illiciaceae chloroplast genomes. **(A)** Number of long-repeat. **(B)** Number of SSR repeat types.

In the analysis of repetitive structures within the Illiciaceae (*I. verum* OR668891*, I. verum* NC_034689*, I. verum* ON357882*, I. verum* OK377288*, I. anisatum* KY085919*, I. henryi* NC_034699*, I. lanceolatum* OL802931) chloroplast genomes, 33 inverted repeat sequences were identified in *I. verum* OR668891 and *I. verum* NC_034689, 30 inverted repeat sequences in *I. verum* ON357882 and *I. verum* OK377288, 28 inverted repeat sequences in *I. anisatum* KY085919, 37 inverted repeat sequences in *I. henryi* NC_034699, and 30 inverted repeat sequences in *I*. *lanceolatum* OL802931. The majority of these repeats range from 9 to 30 base pairs (bp), as detailed in Figure 6A; Supplementary Table S2. The longest repeat, measuring 30 bp, is situated within the large single copy (LSC) region. The distribution of repeats across the genome is as follows: 25 in the LSC, 6 in the small single copy (SSC), and 2 in the inverted repeat (IR) regions. Predominantly, the repeats in intergenic spacers are concentrated in the LSC region, whereas two repeats are located in the SSC region.

The chloroplast genome is highly conserved, allowing SSR primers to be broadly applicable across different species and genera. Through the analysis of SSRs in the Illiciaceae (*I. verum* OR668891*, I. verum* NC_034689*, I. verum* ON357882*, I. verum* OK377288*, I. anisatum* KY085919*, I. henryi* NC_034699*, I. lancolatum* OL802931), 92 SSRs were identified in *I. verum* OR668891 and *I. verum* NC_034689, 89 SSRs in *I. verum* OK377288, 96 SSRs in *I. anisatum* KY085919, 79 SSRs in *I. henryi* NC_034699, and 89 SSRs in *I. lanceolatum* OL802931. The majority of these SSRs were distributed in the large single copy (LSC) and small single copy (SSC) regions, with a subset also present in the inverted repeat (IR) regions. The *I. verum* OR668891 SSRs comprised 79 single nucleotide SSRs (85.8%), and 1 dinucleotide SSRs (1%) (Figure 6B; Table 4). Of these, 12 SSRs were of the C type, while the remainder were A/T types.

**TABLE 4:** Types and amounts of SSRs in the chloroplast genome of *I. verum*.

SSR type	Repeat unit	Amount	Ratio(%)
Mono	A/T	49	60.5
Di	AT	2	7.4
	AG	1	
	TA	1	
	TC	2	
Tri	ACC	1	3.7
	TAT	1	
	AAT	1	
Tetra	AATG	1	6.2
	TTCT	1	
	ATTT	1	
	CATT	1	
	AAAT	1	
Penta	AACTA	1	1.2
c		17	21

### 3.6 Phylogenetic analysis

**FIGURE 7:**
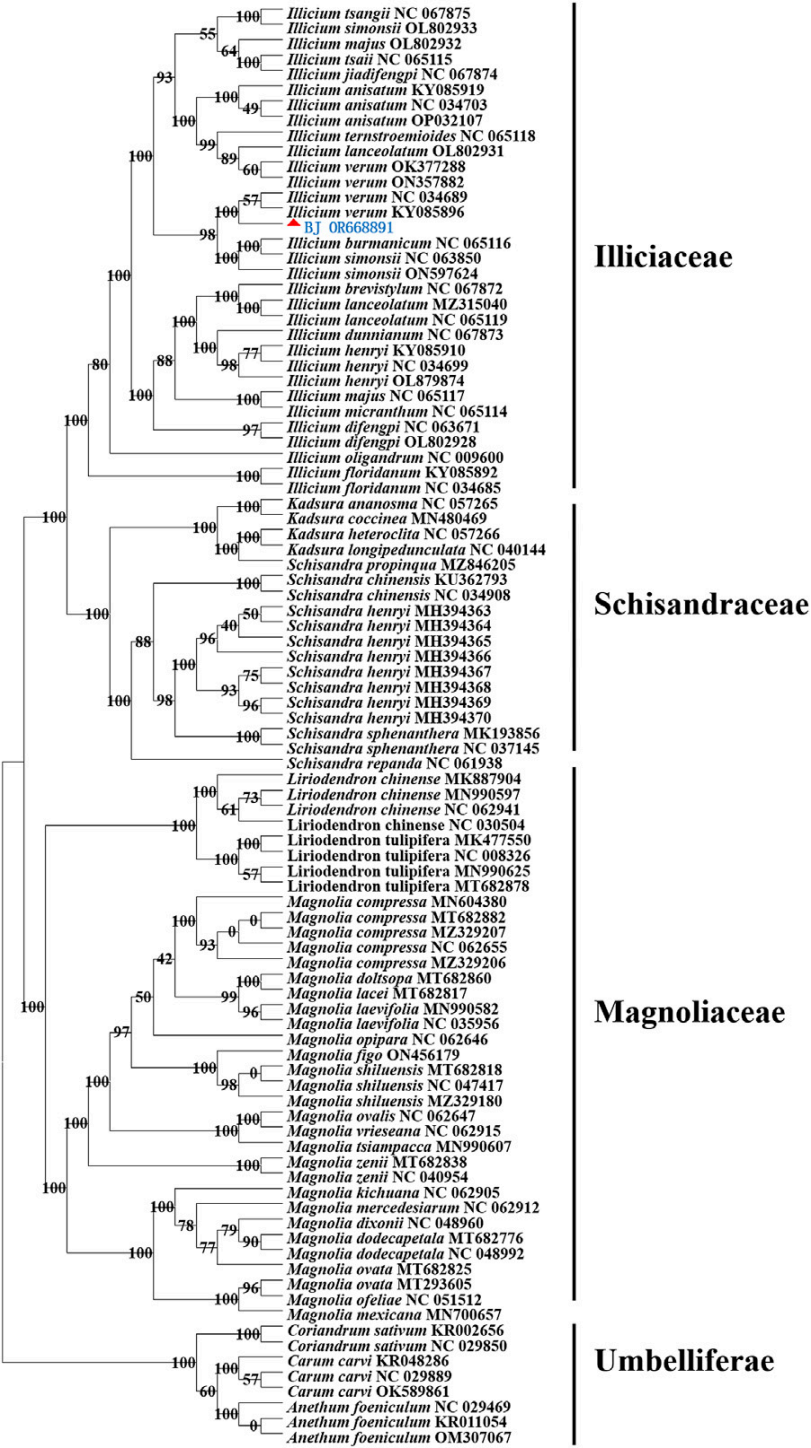
Maximum likelihood (ML) tree of 53 species in different genera of Magnoliaceae and Illiciaceae based on the whole chloroplast genomes. The position of *I. verum* is indicated by “▲.” The cp genomes of other species used in this study were obtained from GenBank. Details for the origin of species were given in Supplementary Table S3.

We found that the Illiciaceae and the Schisandraceae families are grouped together in the same branch. *I. verum* was clustered with *I*. *verum* (KY085896, NC 034689) into a subclade with 100% supported value and *I. verum* was grouped into a terminal branch with Illiciaceae plants such as *I*. *anisatum*, *I*. *lanceolatum*, *I*. *majus*, *I*. *burmanicum*, *I*. *henryi*, *I*. *floridanum and I*. *tsangii*. The family of Schisandriaceae and heteromorphic Schisandriaceae such as *K*. *heteroclita*, *K*. *ananosma*, *S*. *chinensis*, and *S*. *henryi* were clustered into another terminal branch. Meanwhile, the Magnoliaceae plants such as *M*. *ovata*, *M*. *laevifolia*, *M*. *figo*, *M*. *dixonii*, *M*. *zenii*, *M*. *lacei*, *L*. *chinense* and *L*. *tulipifera* formed an independent branch (Figure 7).

## 4 Discussion

The *I. verum* is predominantly found in provinces near the Tropic of Cancer in China, such as Guangxi province, where it is cultivated extensively. According to the latest 2020 edition of the Chinese Pharmacopoeia, the identification of *I. verum* is mainly through trait identification, the color reaction of resorcinol hydrochloric acid, and other methods that include a combination of the above methods.

In the present study, we successfully assembled, annotated, and analyzed the complete chloroplast (cp) genome sequence of *I. verum*. Further analysis focused on its genomic features, guanine-cytosine (GC) content, gene structure, and repetitive sequences. They revealed that the structure and content of *I. verum* are similar to those of other angiosperms. This phenomenon is also widespread in the chloroplast genomes of other angiosperms. The DNA GC content is an important indicator for evaluating the phylogenetic relationships of *I. verum*, and its cpDNA GC content is similar to other species within the Illiciaceae. The DNA GC content in the IR regions is higher than in other regions (LSC, SSC), a phenomenon commonly observed in other plants. The higher DNA GC content in the IR regions is primarily attributed to the rRNA and tRNA genes. The lengths of introns and exons are of significant importance in plant chloroplast genomes. The results indicate that in the chloroplast genome of *I. verum*, only one gene (*rps12*) contains three exons, while two genes (*ycf3* and *clpP*) contain two introns. The *rps12* gene is located at the 5′ end of the LSC region, with the duplicated 3′ end in the IRs region, thus being referred to as a trans-spliced gene. Furthermore, in the codon usage of the *I. verum* chloroplast genome, 2,319 codons encode leucine (10.2%), the most frequently used amino acid, while 288 codons encode tryptophan, the least common amino acid in this species' cp genome. The high frequency of leucine in the chloroplast genomes can be attributed to its substantial requirement in chloroplasts, as leucine plays a significant role in photosynthesis-related metabolism. Comparative analysis of chloroplast genomes from the Illiciaceae, Magnoliaceae and Schisandraceae using mVISTA revealed the similarity in DNA sequences among closely related species. Although the IR regions is more conserved than the SSC and LSC regions in chloroplast genomes, evolutionary events and size variations in chloroplast genomes of different plants can also be attributed to the expansion and contraction of boundaries between the SC and IR regions (Raubeson et al., 2007; Wang et al., 2008; Wang and Messing, 2011; Meng et al., 2018; Zhao et al., 2018; Androsiuk et al., 2020). In this study, the *rps19*, *ndhF*, *ycf1*, and *trnH* genes are located at the boundaries of LSC/IRB, IRB/SSC, SSC/IRA, and IRA/LSC, respectively. Similar to most angiosperms, the *ndhF* and *ycf1* genes of *I*.*verum* chloroplast genome are located at the IR boundary.

SSRs, known as microsatellites, are important repetitive elements throughout the genome, composed of one or a few tandemly repeated nucleotides. They are identical units located in homologous regions but with different unit numbers, playing a significant role in many applications such as species identification, population genetics, and phylogenetic studies (Yang et al., 2011; Jiao et al., 2012; Zhang et al., 2016; Androsiuk et al., 2020; Zhang et al., 2011). SSRs in chloroplast genomes are typically distributed in intergenic regions (Zhao et al., 2015). Based on transcriptome data, Yu Xinghua et al. (Yu et al., 2022) screened SSRs, where dinucleotide and trinucleotide repeat types dominated in the transcriptome data SSRs, with the highest frequency of AG/CT in dinucleotides and AAG/CTT in trinucleotides. Based on the study of chloroplast genes, it was found that the single nucleotide repeats were the most in the SSRs sequence of the chloroplast genome, and this phenomenon was the same in the octagonal chloroplast genome. A total of 92 SSRs were found in the chloroplast genome of *I. verum* OR668891 (Figure 6), most of which were located in the LSC region, followed by the SSC region and the IR region. The most abundant are mononucleotide repeat sequences (85.8%), contributing to A/T richness. These results are consistent with most reported angiosperms (Cui et al., 2019; Gao et al., 2019; Li W. et al., 2019; Li Y. et al., 2019). By comparing repetitive sequences with other plants in the Illiciaceae, it was found that *I*. *verum* (OR668891) has 33 long-repeat sequences and 92 SSRs, which is consistent with *I*. *verum* (NC-034689, KY085896). In contrast, *I*. *verum* (OK377288) has 30 long repetitive sequences and 89 SSRs, and its molecular characteristics are more inclined towards *I. lanceolatum* (OL802931). Comparison of the chloroplast genome SSRs of *I*. *verum* with *I*. *anisatum*, *I*. *henryi*, and *I*. *lanceolatum* revealed certain differences in SSRs among the four species. Currently, there are several complete chloroplast genomes of *I*. *verum* registered in GenBank, with accession numbers ON357882, OK377288, NC_034689, and KY085896. Among these, the sequences ON357882 and OK377288 were submitted by Nanjing Agricultural University in Jiangsu Province, China. Due to limited information available upon registration, it is unclear whether the *I*. *verum* sequences (ON357882 and OK377288) originate from a specific source. Whether a possibility that the *I. verum* sequences published in Jiangsu (ON357882 and OK377288) might be contamination derived from an *I. lanceolatum* sample? Further in-depth and extensive sequencing will be required to verify this. Overall, the chloroplast genome SSRs of *I*. *verum* exhibit rich variation, which may help detect polymorphisms at the intraspecific level and develop species markers for future evolutionary and genetic diversity studies. The DNA barcoding method, as proposed by Hebert (Hebert et al., 2003), can identify species through DNA sequences, *ITS2*, *matK*, *psbA-trnH*, and *rbcL*. However, the identification of closely related species (mainly morphologically confused species within the same genus) still presents various challenges. Therefore, finding suitable DNA markers for these species is essential. The chloroplast genome is commonly used for phylogenetic studies and species identification because its evolutionary rate is slower than that of the nuclear genome. In this study, the analysis of chloroplast genome comparisons among five varieties showed that there are many variable sites in the intergenic regions such as *ndhB-trnH*, *atpH-atpI*, *rpoB-trnC* and *ndhG-ndhI*. The *ndhB-trnH* region consists of one intergenic space (*ndhB-trnH*) and a coding gene (*trnH*); this region is the most variable marker in the Illiciaceae chloroplast genome (Figure 6; Supplementary Figure S1). However, this marker was not extensively used in plant phylogeny and DNA barcoding. The *ndhB-trnH* can be used in subsequent studies. Thus, these regions could potentially serve as different candidate fragments for the identification of Illiciaceae. Overall, these highly differentiated regions provide abundant molecular marker information for the identification and phylogenetic relationship studies of Illiciaceae.

The phylogenetic positions of 53 cp genomes were successfully analyzed. The Illiciaceae, Schisandraceae, and Magnoliaceae were well separated, and plants of the same species were generally grouped into the same clade. However, when comparing *I*. *verum* (OR668891) with *I*. *verum* sequences (such as OK377288, NC 357882) deposited in NCBI, some deficiencies were found in the non-coding regions of different sequences, resulting in the OR668891and OK377288, NC 357882 strains being on separate branches in the evolutionary tree. This is consistent with the analysis results of the above long repeat sequences and SSRs. Due to the limited number of chloroplast genome sequences of *I. verum* published in NCBI, A broader taxon sampling and integration with other molecular makers, e.g., nuclear genes, need to be used to explore the phylogenetic relationship of Illiciaceae. Nevertheless, our phylogenetic studies provide valuable resources for the taxonomic identification, phylogenetics, and evolutionary history research of Illiciaceae.

## 5 Conclusion

The complete chloroplast (cp) genomes of *I. verum* exhibit the characteristic quadripartite structure typical of land plants. Comparative analysis of cp genomes across nine species within the tribe Illiciaceae has demonstrated a high degree of conservation in both genome organization and gene order. Notably, the identification of specific repeated sequences, and simple sequence repeat (SSR) loci, and regions of high variability within these genomes suggests their utility as molecular markers for phylogenetic studies within the tribe. Phylogenetic analysis supports will Illiciaceae is classified as an independent branch. Furthermore, the release of additional cp genome sequences from the genus *Illicium* is anticipated to facilitate the identification of genetic variations, thereby enhancing our understanding of the evolutionary history and interspecific relationships within this group.

## Data Availability

The datasets presented in this study can be found in online repositories. The names of the repository/repositories and accession number(s) can be found below: https://www.ncbi.nlm.nih.gov/genbank/, OR668891.
