# Supplementary material for: The complete chloroplast genome of Illicium verum and comparative analysis with related species from Magnoliaceae and Illiciaceae
Source: Front Genet. 2024 Dec 11;15:1452680. doi: 10.3389/fgene.2024.1452680 (PMC11668812; doi:10.3389/fgene.2024.1452680)
Supplement: Supplementary file 1 [file Table1.docx]

**TABLE S1** Codon-anticodon recognition patterns and codon usage of the *I. verum* chloroplast genome.

| Amino Acid | Total | AA Frequency | Codon | Count | Codon Frequency | RSCU |
| --- | --- | --- | --- | --- | --- | --- |
| L | 2319 | 10.2% | CTA | 321 | 13.8% | 0.831 |
|  |  |  | CTC | 168 | 7.2% | 0.435 |
|  |  |  | CTG | 188 | 8.1% | 0.486 |
|  |  |  | CTT | 474 | 20.4% | 1.226 |
|  |  |  | TTA | 666 | 28.7% | 1.723 |
|  |  |  | TTG | 502 | 21.6% | 1.299 |
| I | 1965 | 8.6% | ATA | 627 | 31.9% | 0.957 |
|  |  |  | ATC | 434 | 22.1% | 0.663 |
|  |  |  | ATT | 904 | 46.0% | 1.38 |
| S | 1763 | 7.8% | AGC | 111 | 6.3% | 0.378 |
|  |  |  | AGT | 347 | 19.7% | 1.181 |
|  |  |  | TCA | 380 | 21.6% | 1.293 |
|  |  |  | TCC | 299 | 17.0% | 1.018 |
|  |  |  | TCG | 152 | 8.6% | 0.517 |
|  |  |  | TCT | 474 | 26.9% | 1.613 |
| G | 1633 | 7.2% | GGA | 637 | 39.0% | 1.56 |
|  |  |  | GGC | 168 | 10.3% | 0.412 |
|  |  |  | GGG | 279 | 17.1% | 0.683 |
|  |  |  | GGT | 549 | 33.6% | 1.345 |
| R | 1420 | 6.2% | AGA | 440 | 31.0% | 1.859 |
|  |  |  | AGG | 173 | 12.2% | 0.731 |
|  |  |  | CGA | 297 | 20.9% | 1.255 |
|  |  |  | CGC | 97 | 6.8% | 0.41 |
|  |  |  | CGG | 99 | 7.0% | 0.418 |
|  |  |  | CGT | 314 | 22.1% | 1.327 |
| A | 1298 | 5.7% | GCA | 363 | 28.0% | 1.119 |
|  |  |  | GCC | 223 | 17.2% | 0.687 |
|  |  |  | GCG | 138 | 10.6% | 0.425 |
|  |  |  | GCT | 574 | 44.2% | 1.769 |
| V | 1289 | 5.7% | GTA | 455 | 35.3% | 1.412 |
|  |  |  | GTC | 170 | 13.2% | 0.528 |
|  |  |  | GTG | 204 | 15.8% | 0.633 |
|  |  |  | GTT | 460 | 35.7% | 1.427 |
| F | 1233 | 5.4% | TTC | 511 | 41.4% | 0.829 |
|  |  |  | TTT | 722 | 58.6% | 1.171 |
| E | 1194 | 5.3% | GAA | 874 | 73.2% | 1.464 |
|  |  |  | GAG | 320 | 26.8% | 0.536 |
| T | 1166 | 5.1% | ACA | 365 | 31.3% | 1.252 |
|  |  |  | ACC | 216 | 18.5% | 0.741 |
|  |  |  | ACG | 124 | 10.6% | 0.425 |
|  |  |  | ACT | 461 | 39.5% | 1.581 |
| K | 1100 | 4.8% | AAA | 797 | 72.5% | 1.449 |
|  |  |  | AAG | 303 | 27.5% | 0.551 |
| N | 1040 | 4.6% | AAC | 245 | 23.6% | 0.471 |
|  |  |  | AAT | 795 | 76.4% | 1.529 |
| P | 981 | 4.3% | CCA | 288 | 29.4% | 1.174 |
|  |  |  | CCC | 220 | 22.4% | 0.897 |
|  |  |  | CCG | 108 | 11.0% | 0.44 |
|  |  |  | CCT | 365 | 37.2% | 1.488 |
| D | 926 | 4.1% | GAC | 201 | 21.7% | 0.434 |
|  |  |  | GAT | 725 | 78.3% | 1.566 |
| Y | 841 | 3.7% | TAC | 177 | 21.0% | 0.421 |
|  |  |  | TAT | 664 | 79.0% | 1.579 |
| Q | 795 | 3.5% | CAA | 584 | 73.5% | 1.469 |
|  |  |  | CAG | 211 | 26.5% | 0.531 |
| H | 597 | 2.6% | CAC | 143 | 24.0% | 0.479 |
|  |  |  | CAT | 454 | 76.0% | 1.521 |
| M | 492 | 2.2% | ATG | 492 | 100.0% | 1 |
| W | 407 | 1.8% | TGG | 407 | 100.0% | 1 |
| C | 268 | 1.2% | TGC | 72 | 26.9% | 0.537 |
|  |  |  | TGT | 196 | 73.1% | 1.463 |

RSCU: relative synonymous codon usage.
