# Supplementary figures and images for: The complete chloroplast genome of Illicium verum and comparative analysis with related species from Magnoliaceae and Illiciaceae
Source: Front Genet. 2024 Dec 11;15:1452680. doi: 10.3389/fgene.2024.1452680 (PMC11668812; doi:10.3389/fgene.2024.1452680)

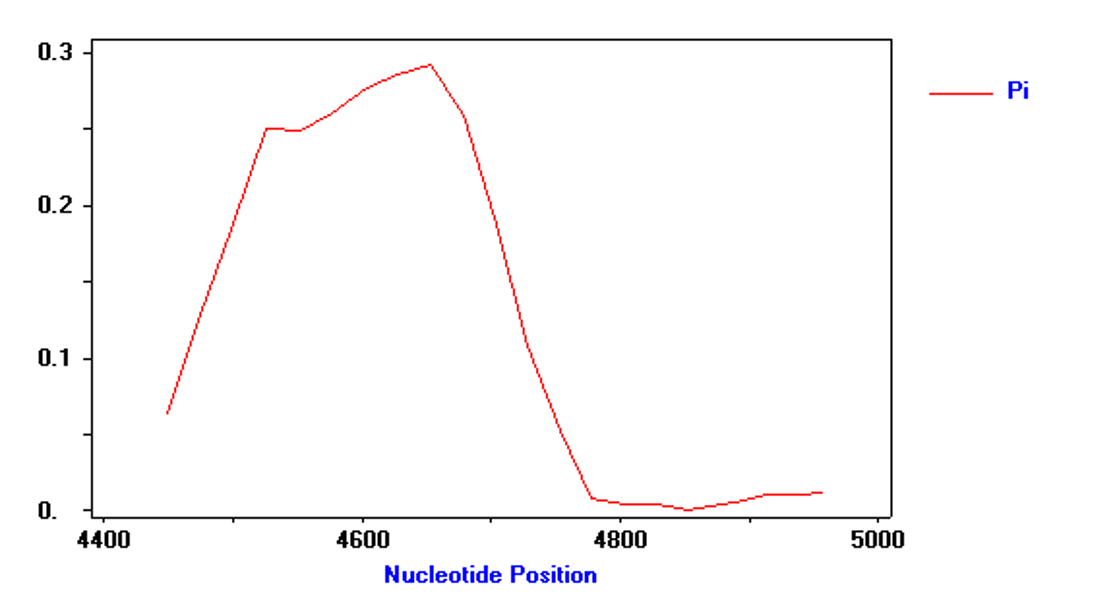

Supplement: Supplementary file 2 [file Image1.tif]
