# Supplementary material for: The complete chloroplast genome of Illicium verum and comparative analysis with related species from Magnoliaceae and Illiciaceae
Source: Front Genet. 2024 Dec 11;15:1452680. doi: 10.3389/fgene.2024.1452680 (PMC11668812; doi:10.3389/fgene.2024.1452680)
Supplement: Supplementary file 3 [file Table2.docx]

**TABLE S2** The lengths of the tandem repeats in the *I. verum* cp genome

| Indices | Period Size | Copy Number | Consensus Size | Consensus pattern | Percent Matches | Percent Indels | Score | A | C | G | T | Entropy (0-2) |
| --- | --- | --- | --- | --- | --- | --- | --- | --- | --- | --- | --- | --- |
| 5047--5076 | 14 | 2.1 | 14 | TCAGTCTTATCTTA | 100 | 0 | 60 | 20 | 23 | 6 | 50 | 1.71 |
| 6538--6562 | 12 | 2.1 | 12 | AGAGAGAAAGAG | 100 | 0 | 50 | 60 | 0 | 40 | 0 | 0.97 |
| 14233--14258 | 13 | 2 | 13 | TACTGGAATAAAA | 100 | 0 | 52 | 53 | 7 | 15 | 23 | 1.67 |
| 20445--20478 | 9 | 3.7 | 9 | TATATCGTA | 84 | 7 | 50 | 32 | 14 | 8 | 44 | 1.76 |
| 33005--33049 | 20 | 2.2 | 20 | TTATAGAGTCTTGTTATAAA | 96 | 0 | 81 | 37 | 4 | 13 | 44 | 1.64 |
| 36777--36832 | 28 | 2 | 28 | TCCCTCCTTAGAACTAGAACAGGATAAA | 100 | 0 | 112 | 39 | 25 | 14 | 21 | 1.91 |
| 37481--37524 | 17 | 2.6 | 16 | ATTTATAATATAATTT | 92 | 7 | 70 | 47 | 0 | 0 | 52 | 1 |
| 37489--37523 | 12 | 2.9 | 12 | TATAATATAATT | 91 | 0 | 52 | 45 | 0 | 0 | 54 | 0.99 |
| 42529--42561 | 16 | 2.1 | 16 | AGCTAAACCATTAAAT | 100 | 0 | 66 | 51 | 18 | 6 | 24 | 1.68 |
| 42531--42567 | 16 | 2.3 | 16 | CTAAACCATTAAATAA | 90 | 0 | 56 | 54 | 16 | 2 | 27 | 1.56 |
| 42795--42836 | 21 | 2 | 21 | ATCCGTATATATAGATAGATG | 95 | 0 | 75 | 35 | 11 | 19 | 33 | 1.88 |
| 52399--52428 | 15 | 2 | 15 | TACCATCATTCCAAC | 93 | 0 | 51 | 33 | 36 | 3 | 26 | 1.73 |
| 53769--53827 | 30 | 1.9 | 31 | TCATTATATATAATATATAATTAATATATTA | 83 | 10 | 77 | 44 | 5 | 0 | 50 | 1.24 |
| 53771--53803 | 13 | 2.7 | 13 | ATTATCATTAAAT | 90 | 9 | 52 | 42 | 6 | 0 | 51 | 1.26 |
| 64992--65022 | 12 | 2.6 | 12 | ATAGTGACATTT | 94 | 0 | 53 | 32 | 6 | 22 | 38 | 1.8 |
| 66482--66513 | 15 | 2.1 | 15 | ATACTTATAATAGAT | 94 | 0 | 55 | 43 | 6 | 9 | 40 | 1.62 |
| 69269--69293 | 11 | 2.3 | 11 | TTACCCATTTT | 100 | 0 | 50 | 20 | 24 | 0 | 56 | 1.43 |
| 69457--69486 | 15 | 2 | 15 | AATTTCTGTATTGAT | 100 | 0 | 60 | 26 | 6 | 13 | 53 | 1.64 |
| 75647--75690 | 21 | 2.1 | 21 | TAAATCCAAGCGAACTTTTCG | 91 | 0 | 70 | 31 | 20 | 13 | 34 | 1.92 |
| 78863--78890 | 9 | 3.1 | 9 | TTCCAGCCC | 100 | 0 | 56 | 10 | 53 | 10 | 25 | 1.67 |
| 86896--86944 | 24 | 2 | 24 | TACGTCCATTCCTACGTAAACCAA | 96 | 0 | 89 | 32 | 30 | 8 | 28 | 1.86 |
| 89416--89453 | 16 | 2.5 | 15 | ATTTATACATAATGC | 87 | 8 | 58 | 42 | 7 | 7 | 42 | 1.63 |
| 89424--89455 | 16 | 2 | 16 | ATAATGCATTTATAAC | 100 | 0 | 64 | 43 | 12 | 6 | 37 | 1.68 |
| 89784--89809 | 13 | 2 | 13 | TCTTTACTTTATT | 100 | 0 | 52 | 15 | 15 | 0 | 69 | 1.2 |
| 96510--96555 | 21 | 2.2 | 21 | TCTTTTTGTCCAAGTCACTTC | 84 | 3 | 58 | 13 | 26 | 4 | 56 | 1.55 |
| 111322--111387 | 18 | 3.4 | 18 | TGTCATTGACATAAGAGA | 69 | 25 | 53 | 36 | 13 | 21 | 28 | 1.91 |
| 114291--114331 | 21 | 2 | 21 | AAATAGCAAATAATATTTATT | 85 | 9 | 57 | 51 | 2 | 7 | 39 | 1.43 |
| 115264--115297 | 16 | 2.1 | 17 | ACTATATGAAATATGAA | 94 | 5 | 61 | 55 | 5 | 11 | 26 | 1.58 |
| 129440--129500 | 27 | 2.3 | 27 | CTTTCCTATTTTCATTTGGTTTATATA | 97 | 0 | 113 | 16 | 16 | 6 | 60 | 1.55 |
| 130159--130197 | 15 | 2.6 | 15 | TCCTCTTTTTTATCT | 91 | 0 | 60 | 5 | 23 | 2 | 69 | 1.21 |
| 130467--130521 | 27 | 2 | 27 | ACTATTAATAGTATTCTCGGTACTAGT | 100 | 0 | 110 | 30 | 14 | 14 | 40 | 1.86 |
| 130467--130535 | 27 | 2.6 | 27 | ACTAGTAATAGTATTCTCGGTACTAGT | 90 | 4 | 95 | 30 | 11 | 15 | 42 | 1.83 |
| 132669--132734 | 18 | 3.4 | 18 | AATGACATCTCTTATGTC | 69 | 25 | 53 | 28 | 21 | 13 | 36 | 1.91 |
