# Supplementary material for: The complete chloroplast genome of Illicium verum and comparative analysis with related species from Magnoliaceae and Illiciaceae
Source: Front Genet. 2024 Dec 11;15:1452680. doi: 10.3389/fgene.2024.1452680 (PMC11668812; doi:10.3389/fgene.2024.1452680)
Supplement: Supplementary file 4 [file Table3.docx]

**TABLE S3** GenBank access numbers for the 53 complete cp genome sequences of the phylogenetic tree

| Family | Organism | Accession |
| --- | --- | --- |
| Illiciaceae | Illicium verum | OR668891 |
| Illiciaceae | Illicium oligandrum | NC 009600 |
| Illiciaceae | Illicium floridanum | NC 034685 |
| Illiciaceae | Illicium floridanum | KY085892 |
| Illiciaceae | Illicium difengpi | OL802928 |
| Illiciaceae | Illicium micranthum | NC 065114 |
| Illiciaceae | Illicium henryi | KY085910 |
| Illiciaceae | Illicium dunnianum | NC 067873 |
| Illiciaceae | Illicium brevistylum | NC 067872 |
| Illiciaceae | Illicium simonsii | ON597624 |
| Illiciaceae | Illicium simonsii | NC 063850 |
| Illiciaceae | Illicium burmanicum | NC 065116 |
| Illiciaceae | Illicium verum | KY085896 |
| Illiciaceae | Illicium anisatum | OP032107 |
| Illiciaceae | Illicium anisatum | KY085919 |
| Illiciaceae | Illicium ternstroemioides | NC 065118 |
| Illiciaceae | Illicium lanceolatum | OL802931 |
| Illiciaceae | Illicium verum | OK377288 |
| Illiciaceae | Illicium tsaii | NC 065115 |
| Illiciaceae | Illicium jiadifengpi | NC 067874 |
| Illiciaceae | Illicium majus | OL802932 |
| Illiciaceae | Illicium simonsii | OL802933 |
| Illiciaceae | Illicium tsangii | NC 067875 |
| Schisandraceae | Kadsura longipedunculata | NC 040144 |
| Schisandraceae | Kadsura heteroclita | NC 057266 |
| Schisandraceae | Kadsura ananosma | NC 057265 |
| Schisandraceae | Kadsura coccinea | MN480469 |
| Schisandraceae | Schisandra repanda | NC 061938 |
| Schisandraceae | Schisandra chinensis | KU362793 |
| Schisandraceae | Schisandra henryi | MH394370 |
| Schisandraceae | Schisandra sphenanthera | NC 037145 |
| Magnoliaceae | Liriodendron chinense | NC 030504 |
| Magnoliaceae | Liriodendron chinense | MK887904 |
| Magnoliaceae | Liriodendron tulipifera | MK477550 |
| Magnoliaceae | Magnolia mexicana | MN700657 |
| Magnoliaceae | Magnolia ofeliae | NC 051512 |
| Magnoliaceae | Magnolia ovata | MT293605 |
| Magnoliaceae | Magnolia dixonii | NC 048960 |
| Magnoliaceae | Magnolia dodecapetala | NC 048992 |
| Magnoliaceae | Magnolia kichuana | NC 062905 |
| Magnoliaceae | Magnolia mercedesiarum | NC 062912 |
| Magnoliaceae | Magnolia ovata | MT682825 |
| Magnoliaceae | Magnolia zenii | NC 040954 |
| Magnoliaceae | Magnolia tsiampacca | MN990607 |
| Magnoliaceae | Magnolia vrieseana | NC 062915 |
| Magnoliaceae | Magnolia ovalis | NC 062647 |
| Magnoliaceae | Magnolia shiluensis | MZ329180 |
| Magnoliaceae | Magnolia figo | ON456179 |
| Magnoliaceae | Magnolia opipara | NC 062646 |
| Magnoliaceae | Magnolia compressa | MZ329206 |
| Magnoliaceae | Magnolia doltsopa | MT682860 |
| Magnoliaceae | Magnolia lacei | MT682817 |
| Magnoliaceae | Magnolia laevifolia | NC 035956 |
